# Photocatalytic Hydrogen Production Driven by Solar Energy: Performance Under Central European Climatic Conditions

**DOI:** 10.3390/ijms27093822

**Published:** 2026-04-25

**Authors:** Wiktoria Kluba, Karol Hauza, Anna Lewandowska-Andralojc

**Affiliations:** 1 Faculty of Chemistry, Adam Mickiewicz University, Uniwersytetu Poznanskiego 8, 61-614 Poznan, Poland; 2 Center for Advanced Technologies, Adam Mickiewicz University, Uniwersytetu Poznanskiego 10, 61-614 Poznan, Poland

**Keywords:** photocatalysis, solar energy conversion, eosin Y, graphene oxide

## Abstract

Photocatalytic hydrogen production represents a promising approach for sustainable fuel generation, particularly when driven by solar irradiation. In this study, a photocatalytic system composed of eosin Y, cobalt sulfate, triethanolamine, and graphene oxide was investigated for hydrogen evolution. The optical and structural properties of the system components were characterized using UV–Vis spectroscopy, FT-IR spectroscopy, Raman spectroscopy, and atomic force microscopy. Photocatalytic activity was evaluated under both artificial light sources (halogen lamp, xenon lamp, and LED 505 nm) and natural sunlight in order to assess system performance under realistic environmental conditions. The addition of graphene oxide significantly enhanced hydrogen production, resulting in an approximately 4-fold increase compared to the three-component system without graphene oxide. Solar-driven experiments conducted over one year demonstrated efficient hydrogen evolution under a wide range of weather and irradiance conditions. Importantly, based on combined experimental and meteorological data, it is shown that high photocatalytic performance is achievable for a substantial fraction of the year, with approximately 55% of days expected to provide at least 80% of the maximum hydrogen production efficiency under Central European climatic conditions. These findings highlight the strong potential of the investigated four-component system for efficient hydrogen generation using low amounts of catalytic material and without external electrical energy input. Overall, the system shows promising performance for solar-driven hydrogen production under real-world solar irradiation conditions.

## 1. Introduction

While recent decades have seen rapid technological development driven by human curiosity and societal demands, the natural environment has concurrently experienced the escalating impacts of global warming and climate change. These effects are largely driven by energy production based on the combustion of fossil fuels and the intensive exploitation of natural resources [1]. In July 2024, three of the hottest days in recorded history were observed, while carbon dioxide emissions reached a record-high level, highlighting the urgent need for a transition to cleaner energy sources [2].

**Figure 1:**
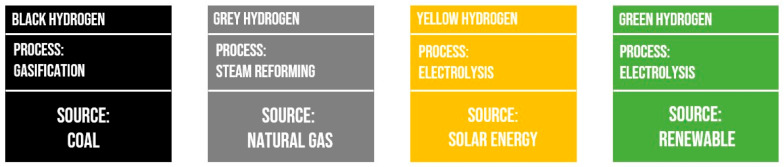
Classification of hydrogen according to the production method.

Hydrogen has emerged as a key environmentally friendly fuel due to its high energy density and the potential for near-zero emissions, since water is the only product of its combustion [3]. Nevertheless, the environmental impact of hydrogen strongly depends on its production pathway [4,5]. At present, most industrial hydrogen is generated via steam reforming of methane or carbon monoxide, processes that are associated with greenhouse gas emissions [6]. Consequently, such hydrogen is classified as grey or black, undermining its role as a truly sustainable energy vector (Figure 1) [7].

Although water electrolysis is currently the most mature low-emission route for green hydrogen production [8], its large-scale economic deployment is still limited by electricity demand and system losses, including electrode overpotentials and heat generation [9]. Photocatalytic hydrogen production also faces significant challenges, such as side reactions, charge-carrier recombination, thermal dissipation, and still relatively low overall solar-to-hydrogen conversion efficiencies. Nevertheless, because it can in principle use solar radiation directly as the energy input, it is being intensively explored as a complementary route for sustainable hydrogen production.

Recent data indicate a rapid expansion of renewable energy sources, particularly wind and solar. In 2024, their generation increased by 16%, with solar power nearly doubling over the past two years [10]. Within Europe, fossil fuel consumption has generally declined over the past decade, reflecting ongoing decarbonization efforts. Major economies such as Germany, France, and the United Kingdom have reduced fossil energy use by over 20% since 2017, while Central and Eastern European countries have experienced more moderate reductions. Poland’s share of renewable energy reached 7.9% in 2024, up from near-zero levels in the early 1990s, highlighting both significant advancement and persistent challenges associated with transitioning toward low-carbon energy systems under Central European climatic and infrastructural conditions. Notably, hydrogen remains absent from power sector decarbonization metrics, underscoring its current status as an emerging rather than established energy carrier [11].

In the face of the global pursuit of sustainable energy sources, hydrogen production via solar-driven photocatalysis emerges as a particularly promising alternative. Unlike approaches that rely on electrical energy, the direct use of sunlight enables a significant reduction in the process’s carbon footprint, making it environmentally more advantageous. This strategy not only aligns with the broader shift toward renewable energy sources but is also supported by local climatic conditions. Long-term meteorological observations from the Institute of Meteorology and Water Management (IMGW) indicate that Poland experiences a moderate annual sunshine duration of approximately 1600–1800 h, with pronounced seasonal and regional variability [12]. Such conditions suggest that solar-driven photocatalytic hydrogen production is a feasible and sustainable pathway under Central European climates, paving the way for its integration into regional clean energy systems.

Unlike most previous studies relying on artificial light sources such as xenon lamps or LEDs, this work evaluates hydrogen production under natural sunlight. The photocatalytic system, previously examined under LED irradiation, consisted of eosin Y (EY), cobalt sulfate, triethanolamine (TEOA), graphene oxide (GO), and is here investigated under natural sunlight [13]. This system was selected as a model photocatalytic system because of its literature-documented activity, simple composition, good reproducibility, and suitability for long-term outdoor studies under natural sunlight. Eosin Y is a dye widely employed as a photosensitizer in photocatalytic hydrogen production, while graphene oxide is a well-studied two-dimensional material reported to act as an electron mediator and enhance hydrogen evolution efficiency [14,15,16,17,18,19]. In this system, CoSO_4_ serves as a pre-catalyst for hydrogen production. Photocatalytic experiments were performed under real sunlight conditions, providing a more realistic assessment of system performance compared to artificial irradiation (Xe lamps, LEDs). Preliminary observations indicate that effective hydrogen generation can be achieved even under moderate sunlight conditions, which is particularly relevant for Central European climate.

## 2. Results

### 2.1. Characterization of System Components

**Figure 2:**
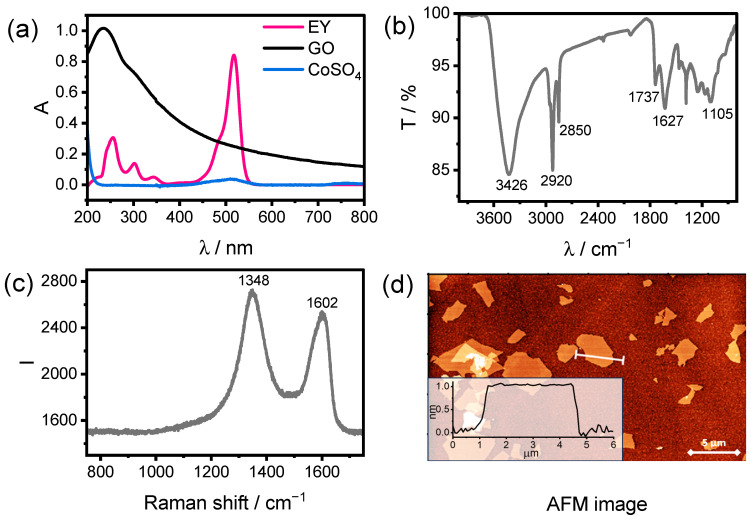
(**a**) Absorption spectra of aqueous solution of EY (10 µM) (pink), GO (13.3 µg/mL) (black) and CoSO_4_ (11.6 µg/mL) (blue). (**b**) FT-IR spectrum of GO. (**c**) Raman spectrum of GO. (**d**) AFM image of graphene oxide with height profile of a monolayer region.

The optical absorption characteristics were first examined by UV–Vis spectroscopy, as light harvesting is a key factor governing the photocatalytic performance of the system. UV–Vis spectra of individual component solutions are displayed in Figure 2a, while spectra of the complete reaction mixtures recorded at the actual concentrations used during catalysis are provided in Figure S1. EY (eosin Y), acting as the photosensitizer, exhibits a strong absorption band in the visible region with a maximum at 516 nm, which is consistent with its ability to efficiently harvest visible light and initiate photoinduced electron transfer processes. EY concentration for photocatalytic tests was selected based on prior optimization studies [20]. At 0.5 mM, the EY concentration ensures very efficient absorption of light in the spectral range where EY absorbs. In contrast, CoSO_4_ shows only a faint pink coloration in solution and does not display any pronounced absorption features. This indicates that cobalt sulfate does not compete with EY for photon absorption and therefore does not hinder the photoexcitation process. The UV–Vis spectrum of GO exhibits a characteristic absorption maximum in the ultraviolet region at approximately 230 nm, which can be assigned to π–π electronic transitions within the sp^2^-hybridized carbon domains. Additionally, a less intense shoulder extending toward ~300 nm is observed and is commonly associated with n–π* transitions related to oxygen-containing functional groups [21]. This absorption behavior is typical for GO and confirms the presence of disrupted conjugated carbon networks resulting from oxidative functionalization.

To further elucidate the surface chemistry of GO, FT-IR spectroscopy was employed (Figure 2b). The FT-IR spectrum reveals multiple vibrational bands characteristic of oxygen functionalities on the GO surface. A broad absorption band centered around 3400 cm^−1^ is attributed to O–H stretching vibrations, indicating the presence of hydroxyl groups. Distinct bands observed at approximately 2920 and 2850 cm^−1^ are attributed to C–H stretching vibrations of aliphatic groups, indicating the presence of residual hydrocarbon moieties on the graphene oxide surface. The peak observed at 1737 cm^−1^ corresponds to C=O stretching vibrations of carbonyl and carboxyl groups, while the band near 1627 cm^−1^ is associated with skeletal C=C vibrations of the graphitic framework, partially overlapping with O–H bending modes of adsorbed water. Additional band at 1105 cm^−1^ is assigned to C–O–C stretching vibrations, respectively, confirming the presence of epoxy and alkoxy functionalities [22,23].

Raman spectroscopy was employed to further assess the structural characteristics of GO (Figure 2c). The Raman spectrum displays two broad and intense bands of comparable intensity: the D band located at approximately 1348 cm^−1^ and the G band centered around 1602 cm^−1^. The D band is associated with defect-activated vibrational modes of aromatic rings, reflecting the presence of structural disorder induced by oxidation, whereas the G band corresponds to the in-plane stretching vibrations of sp^2^-hybridized carbon atoms within the graphitic domains. The relative intensities and positions of these bands are characteristic of graphene oxide and confirm the disrupted sp^2^ carbon network resulting from oxygen functionalization [24].

The morphology of GO was investigated by atomic force microscopy (AFM). AFM images reveal the presence of well-exfoliated GO sheets with lateral dimensions ranging from approximately 0.2 to 5 μm (Figure 2d). The measured thickness of individual sheets is about 0.9 nm, which is consistent with single-layer graphene oxide reported in the literature [25]. These observations confirm the successful exfoliation of graphene oxide into predominantly monolayer sheets, providing a high surface area and abundant accessible functional groups.

### 2.2. Photocatalytic Activity

The photocatalytic performance of the EY–TEOA–CoSO_4_–GO system was evaluated under both artificial irradiation and natural sunlight to assess its practical applicability. Solar-driven experiments were conducted, under varying light intensities, temperatures, and weather conditions. In parallel, control experiments were performed under artificial laboratory irradiation using identical reaction conditions, including the same sample volumes and concentrations of all components, to enable direct comparison with experiments conducted under natural sunlight.

#### 2.2.1. Photocatalysis with Artificial Light Irradiation

The hydrogen production rate was first evaluated under irradiation (λ > 400 nm) in a photocatalytic system consisting of EY as a photosensitizer, cobalt salt CoSO_4_ as a pre-catalyst and TEOA as a sacrificial donor in the reaction system. No significant amounts of hydrogen production were detected in the absence of either irradiation or the photosensitizer EY, indicating that hydrogen generation proceeded via a photochemical reaction. Similarly, control experiments performed without CoSO_4_ showed no appreciable hydrogen evolution, confirming the essential role of the cobalt catalyst in the reaction system.

**Figure 3:**
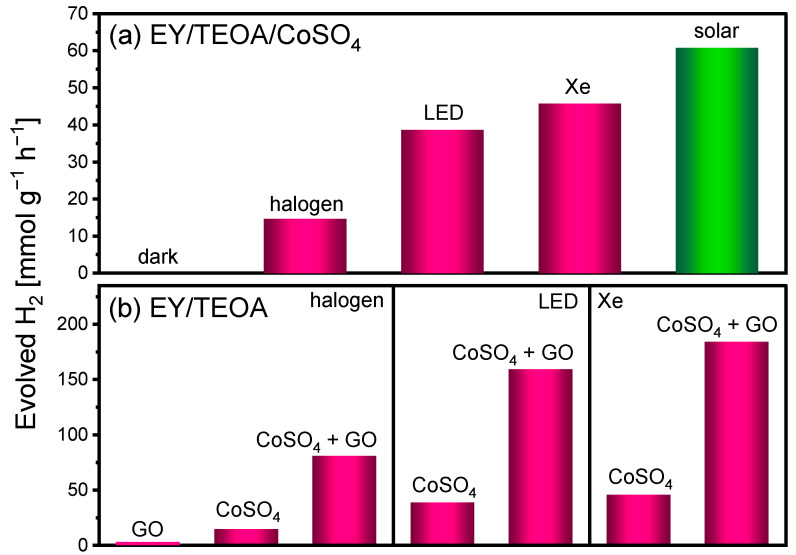
(**a**) Dependence of hydrogen production on the type of irradiation source (three-component system) (**b**) Influence of graphene oxide addition and irradiation source on hydrogen evolution efficiency. Conditions: [EY] = 0.5 mM, [Co^2+^] = 0.7 mg, [TEOA] = 0.2 M, [GO] = 0.8 mg, pH 10.2, T = 25 °C (artificial sources), irradiation time: 1 h.

The samples were irradiated using three artificial light sources, namely a halogen lamp, a xenon lamp, and a 505 nm LED. Hydrogen production was observed under all three light sources. The observed differences in hydrogen evolution should be considered in terms of both the incident light intensity and the spectral overlap between the emission profile of the light source and the absorption band of EY. The incident irradiance values measured at the reactor position were 75 W m^−2^ for the halogen lamp, 150 W m^−2^ for the LED source, and 450 W m^−2^ for the xenon lamp. Irradiation with the LED lamp resulted in approximately 2.7-fold higher hydrogen evolution compared to the halogen lamp, while the xenon lamp yielded more than three times higher hydrogen production relative to the halogen source (Figure 3a). The high activity under LED irradiation can be attributed to its emission centered at 505 nm, which closely matches the absorption maximum of EY and thus allows efficient and selective excitation of the photosensitizer, despite its lower incident irradiance than that of the xenon lamp. In contrast, the higher hydrogen evolution observed under xenon irradiation relative to the halogen lamp likely arises from the combined effect of higher incident irradiance and a more favorable spectral distribution in the wavelength range relevant to EY absorption.

In addition, a preliminary experiment performed under natural sunlight using the same reference system revealed a hydrogen evolution rate approximately 1.3 times higher than that obtained under xenon lamp irradiation Importantly, the irradiance under the applied solar conditions was the highest among all light sources used in this study, exceeding that of the xenon lamp, LED source, and halogen lamp. Therefore, the higher hydrogen evolution observed under sunlight can be attributed, at least in part, to the higher incident solar irradiance. At the same time, the overall performance under sunlight is also influenced by the broad spectral distribution of solar radiation, only a fraction of which overlaps with the absorption range of EY and can be directly utilized for photosensitizer excitation. Despite this limitation, the results clearly demonstrate the strong potential of the investigated system for solar-driven hydrogen production and motivated further studies aimed at a detailed evaluation of the dependence of hydrogen evolution on natural sunlight intensity.

To further enhance the hydrogen production efficiency, we explored hydrogen evolution in the same photocatalytic system with the addition of GO. Numerous studies demonstrated that graphene-based materials can significantly improve photocatalytic hydrogen production [26,27]. To evaluate whether graphene oxide could act as an independent hydrogen evolution catalyst, a control experiment was performed under irradiation using a system in which CoSO_4_ was omitted while graphene oxide was retained. Under these conditions, hydrogen evolution remained at nearly zero levels, demonstrating that graphene oxide does not exhibit intrinsic catalytic activity toward hydrogen production (Figure 3b). These findings confirm that CoSO_4_ is essential for catalytic activity and act as a precursor of the catalytically active phase formed under irradiation. Most likely, Co^2+^ ions undergo in situ photoreduction to cobalt-based nanoparticles, which subsequently anchor onto the surface of graphene oxide, forming a stable and well-dispersed photocatalytic system. Graphene oxide thus functions as a support that promotes cobalt dispersion, enhances particle stability, and limits their aggregation [20,28]. Consistent with this interpretation, the introduction of GO into the system leads to a pronounced enhancement of hydrogen production, regardless of the irradiation source. Notably, the addition of only 0.8 mg of GO per sample results in a substantial enhancement of hydrogen evolution. Compared to the corresponding three-component system without graphene oxide, hydrogen production increased by approximately fourfold under xenon lamp and under LED irradiation. For clarity, the hydrogen evolution rates were normalized with respect to the mass of the catalytic component present in the system. In the three-component system, this corresponds solely to the mass of cobalt ions, whereas in the four-component system the total mass of both cobalt and graphene oxide was taken into account. These results demonstrate that even a small amount of graphene oxide can significantly influence on the overall hydrogen production efficiency. The marked improvement in photocatalytic activity after incorporation of graphene oxide can be attributed to its dual role as an efficient electron acceptor and charge mediator, facilitating charge separation and suppressing recombination processes, as well as a support that promotes cobalt dispersion, enhances particle stability, and limits their aggregation.

**Figure 4:**
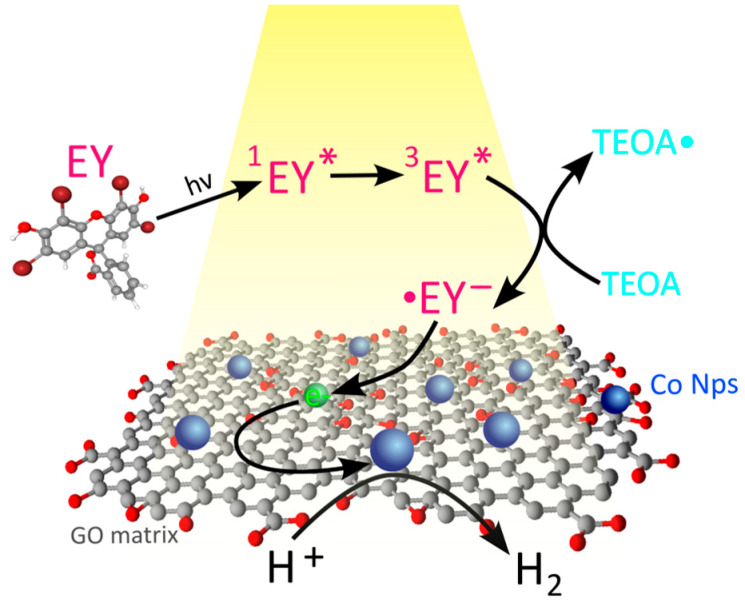
Schematic illustration of the general mechanism proposed for the photocatalytic hydrogen evolution process.

This interpretation is consistent with the reaction mechanism proposed in previous report, in which graphene oxide was shown to act as an efficient electron-accepting and charge-mediating component [13]. Following photoexcitation of eosin Y and reductive quenching by TEOA, the resulting EY radical anion transfers electrons to the graphene oxide sheets, promoting efficient charge separation and suppressing recombination. The accumulated electrons are then transferred to the cobalt precursor, resulting in its in situ photoreduction and the formation of Co nanoparticles anchored on the GO surface. These photogenerated Co NPs likely constitute the catalytically active sites responsible for hydrogen evolution. Importantly, graphene oxide does not affect the primary photoexcitation of the photosensitizer but improves the efficiency of subsequent electron-transfer processes (Figure 4).

#### 2.2.2. Recyclability of the System

The durability and reusability of a photocatalytic system are crucial factors for its practical application. As shown in Figure 4, hydrogen production plateaued after 2 h of irradiation. This was accompanied by a noticeable color change (Figure S2), which was attributed to the photodegradation of photosensitizer. EY, being a brominated derivative of fluorescein, is known to undergo photodegradation/debromination under irradiation, which explains the observed change in the reaction mixture color [29].

**Figure 5:**
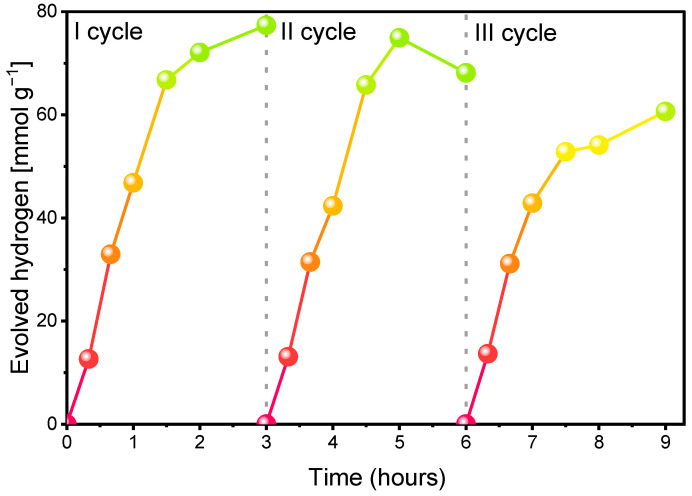
The amount of evolved H_2_ by EY/Co^2+^/GO/TEOA system in three reaction runs. The irradiation was conducted for 9 h, with periodic interruptions every 3 h and fresh EY, TEOA repletion. Conditions: [EY] = 0.5 mM, [Co^2+^] = 0.7 mg, [TEOA] = 0.2 M, [GO] = 0.8 mg, pH 10.2, T = 25 °C, irradiation time: 3 h.

To assess the stability of the catalytic components, a three-cycle recycling experiment was performed using the same batch of CoSO_4_ and GO, recovered by centrifugation. In each cycle, fresh portions of EY and TEOA were added, and the system was irradiated for 3 h. The photocatalytic activity was monitored by measuring hydrogen evolution over time (Figure 5). The highest activity was observed during the first cycle. In the second and third cycles, hydrogen production showed only a modest decrease. Quantitatively, after 3 h of irradiation, the hydrogen evolution on the second and third cycle corresponded to 88% and 78% of the activity recorded on the first cycle, respectively. The decline in activity observed during a single cycle is mainly related to the gradual photodegradation of EY, rather than to irreversible deactivation of the catalyst. Notably, replenishment of EY in the presence of the recovered solid catalyst, composed of GO with adsorbed Co nanoparticles, largely restored the photocatalytic performance. Therefore, the gradual decrease in activity over subsequent cycles can be attributed predominantly to catalyst loss during the recovery process, since repeated centrifugation and redispersion inevitably caused partial loss of GO sheets decorated with Co nanoparticles. When the comparison is made after 1 h of irradiation, the results indicate very good cycle reproducibility of the system. The activity measured on the second and third cycle corresponded to 90% and 92% of the first cycle value, respectively, demonstrating good short-term reproducibility and operational stability of the catalytic system. For this reason, 1 h irradiation was selected as the standard time in solar-driven experiments. Moreover, considering that the experiments were performed under natural sunlight, shorter irradiation times minimize the influence of fluctuating weather conditions. Prolonged exposure (2–3 h) increases the likelihood of changes in solar intensity, temperature, and cloud coverage, which may additionally affect the measured photocatalytic activity.

#### 2.2.3. Photocatalysis with Direct Sunlight Irradiation

**Figure 6:**
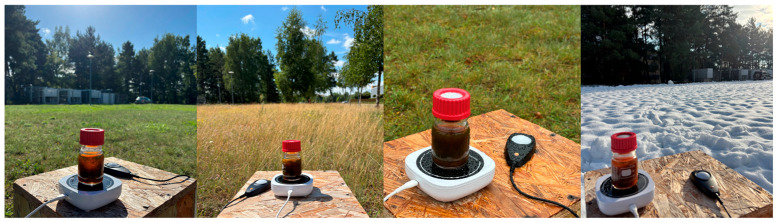
Photographs illustrating photocatalytic experiments conducted under different weather conditions and seasons. From left to right: spring (clear sky), summer (partly cloudy), autumn (rainy), and winter (snowy with sunshine), illustrating the variability of natural sunlight conditions during outdoor irradiation.

To evaluate the practical applicability of the system, photocatalytic hydrogen production experiments were performed under direct sunlight. A photograph of the experimental setup is presented in Figure 6. The reactor consisted of a 100 mL Duran glass bottle placed on a portable magnetic stirrer to ensure continuous mixing during irradiation. Solar irradiance values were obtained from the ERA5 global reanalysis dataset for the specified geographic location and the corresponding irradiation time of each experiment. The solar-driven experiments were conducted over a period of approximately one year, allowing the system to be tested under a wide range of seasonal and weather conditions (Figure 6). To ensure experimental consistency and comparability, all measurements were performed at the same outdoor location, without changing orientation of the reactor throughout the study. Furthermore, irradiation was consistently carried out within the same time window (11:30–12:30), corresponding to the period around solar noon, when the angle of solar incidence is highest and sunlight intensity reaches its daily maximum. Such an experimental design enabled the photocatalytic system to be evaluated under realistic outdoor conditions, providing a reliable assessment of its performance for solar-driven hydrogen production.

**Figure 7:**
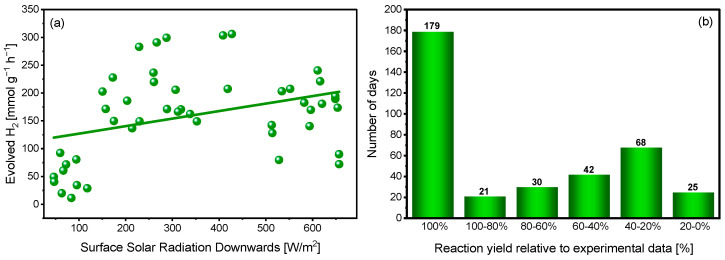
(**a**) Correlation between solar radiation and the amount of evolved hydrogen. The plot compiles all experimental data points obtained during the photocatalytic measurements with direct sunlight irradiation. Conditions: [EY] = 0.5 mM, [Co^2+^] = 0.7 mg, [TEOA] = 0.2 M, [GO] = 0.8 mg, pH 10.2 irradiation time: 1 h. (**b**) Meteorological statistics of sunny days characterized by optimal conditions for hydrogen production in the studied system.

Figure 7a illustrates the relationship between surface solar radiation and the amount of hydrogen evolved during 1 h of irradiation under natural sunlight. A clear linear relationship between solar radiation intensity and hydrogen evolution is not observed, indicating that higher irradiance does not necessarily translate into proportionally higher hydrogen production. This can be considered a favorable outcome, as it suggests that efficient hydrogen generation is achievable even without extreme sunlight conditions. At the same time, a noticeable scatter of experimental data is present, which can be attributed to fluctuations in environmental parameters such as temperature, cloud coverage, and spectral variations of natural sunlight. Moreover, excessively high irradiance may be detrimental to the overall photocatalytic efficiency, as it can enhance parasitic photoinduced pathways, accelerate the photodegradation of EY, and promote other non-productive processes competing with hydrogen evolution. This behavior also suggests that light absorption is not the rate-determining step under these conditions; instead, the system may become partially limited by subsequent processes such as charge recombination, electron transfer, or mass transport. Importantly, high hydrogen production rates are observed within the moderate radiation range of 200–400 W/m^2^, which corresponds well to typical outdoor conditions in Central Europe, where irradiance rarely reaches the standard 1000 W/m^2^ (1 sun) due to geographical latitude and solar elevation. This observation further supports the practical applicability of the investigated system for solar-driven hydrogen production under real environmental conditions. Extrapolation of the regression line fitted to all experimental data points indicates that at the maximum recorded radiation of approximately 700 W/m^2^, the predicted hydrogen production reaches 208 mmol g^−1^ h^−1^. Importantly, this value results directly from the regression model rather than from a single experimental measurement. This finding has practical significance, as it demonstrates that efficient hydrogen evolution does not require peak solar irradiance and can be sustained under sunlight levels commonly encountered in real outdoor environments.

Based on experimental data and meteorological statistics for obtained from the ERA5 global reanalysis dataset (Climate Data Store) for the latitude and longitude of the measurement site, the distribution of days within a year that enable efficient photocatalytic hydrogen production was assessed (Figure 7b). The day of October 20, on which a maximum hydrogen production yield of 306 mmol g^−1^ was achieved, was taken as the reference point and defined as 100% efficiency. Subsequently, the total solar irradiance was calculated for a fixed 6 h period (09:00–15:00) on that day. This time window was intentionally selected to provide a common reference interval available throughout the year, thereby minimizing the influence of seasonal variability in day length and allowing direct comparison between different days. It was assumed that the irradiance sum recorded during this 6 h interval on the reference day corresponds to the experimentally observed maximum hydrogen production level.

Based on this criterion, the number of days in a year for which the solar irradiance within the same 6 h period meets or exceeds a given fraction of the reference irradiance threshold was determined. The analysis indicates that for approximately 55% of days in a year, hydrogen production efficiency during this fixed 6 h interval would reach at least 80% of the maximum value. Interestingly, for fewer than 6% of days, the efficiency would fall within the 0–20% range (Figure 7b). It should also be noted that the identified number of days does not imply the occurrence of 179 exceptionally sunny days per year. Experimental results indicate that the maximum hydrogen production was not necessarily obtained under the highest possible solar irradiance. This suggests that, above a certain irradiation threshold, further increases in solar intensity do not lead to a proportional increase in hydrogen production, likely due to saturation effects or other limiting kinetic factors in the photocatalytic system. Therefore, a relatively large number of days may still provide irradiation conditions sufficient to sustain near-maximum hydrogen production, even if they would not be classified as exceptionally sunny in a meteorological sense. Importantly, our analysis refers specifically to the 09:00–15:00 period and therefore represents the minimum performance expected during a standardized daylight window that is present year-round. In seasons with longer daylight hours, especially in spring and summer, the total daily hydrogen production may be even higher, since irradiation outside the analyzed 6 h interval can additionally contribute to photocatalytic activity. Therefore, the presented results should be interpreted as a conservative estimate of daily performance rather than the upper limit of hydrogen production under real outdoor conditions.

These results suggest that, under the adopted assumptions, photocatalytic hydrogen production could operate at relatively high efficiency for a significant fraction of the year. This indicates favorable climatic conditions for solar-driven hydrogen generation in the analyzed location. However, it should be noted that the model is based solely on solar irradiance and does not account for other environmental factors, which may influence the actual performance of the photocatalytic process.

While no strong increase in hydrogen production with rising light intensity is observed, the overall weak correlation indicates that solar irradiance is not the sole factor governing photocatalytic performance. Other environmental and material-specific variables likely contribute to the observed variability in hydrogen evolution. These may include ambient temperature, wind conditions affecting heat dissipation, transient cloud coverage, and short-term fluctuations in solar spectrum composition. Therefore, while light intensity is the dominant driving factor, photocatalytic efficiency under outdoor conditions is governed by a combination of meteorological variables.

**Figure 8:**
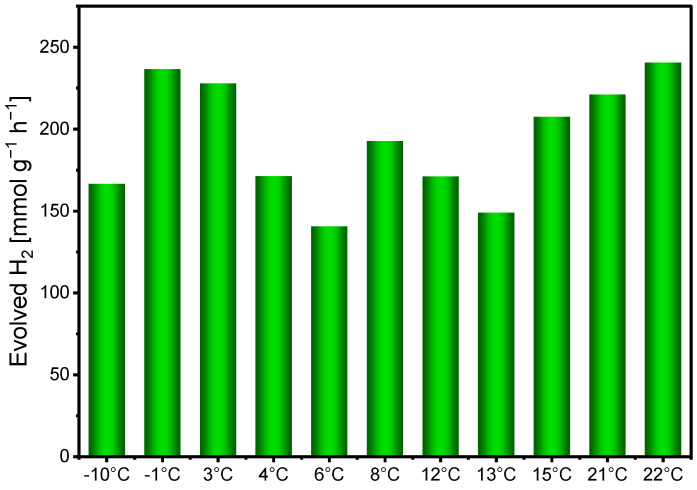
Dependence of the amount of evolved hydrogen on temperature under conditions of high solar irradiance. Conditions: [EY] = 0.5 mM, [Co^2+^] = 0.7 mg, [TEOA] = 0.2 M, [GO] = 0.8 mg, pH 10.2, irradiation time: 1 h.

Following the assessment of solar irradiance, the influence of ambient temperature on hydrogen evolution was investigated (Figure 8). The bar chart indicates that hydrogen production remains relatively stable over the temperature range from −10 °C to 30 °C. No clear monotonic trend between temperature and hydrogen evolution can be observed across the examined range. Notably, measurable hydrogen production occurs even at sub-zero temperatures, demonstrating that the photocatalytic process can proceed under cold outdoor conditions. Although slightly higher hydrogen production is occasionally observed at elevated temperatures, the effect is not systematic. This suggests that, within the examined range, the system is not strongly temperature-dependent and can operate efficiently under typical outdoor conditions, including cool or moderately cold environments. Such temperature tolerance enhances the practical applicability of the photocatalytic system in diverse climatic regions. The relatively weak temperature dependence is consistent with the fact that the photocatalytic process is primarily driven by photon absorption rather than thermal activation.

## 3. Discussion

The results demonstrate that the EY–Co^2+^–TEOA–GO photocatalytic system exhibits efficient hydrogen evolution under both artificial light sources and natural sunlight. Spectroscopic characterization confirmed the expected structural and optical properties of the system components. In particular, eosin Y shows strong absorption in the visible region with a maximum around 516 nm, enabling efficient harvesting of visible light and initiation of the photocatalytic process. Control experiments confirmed that GO does not exhibit intrinsic catalytic activity toward hydrogen evolution. Instead, its role is associated with enhanced charge separation and facilitated electron transfer, effectively suppressing charge recombination. The incorporation of graphene oxide led to a substantial increase in hydrogen evolution efficiency, resulting in a 4–6-fold enhancement compared with the three-component system. Recycling experiments demonstrated good stability of the catalytic components. During consecutive catalytic cycles, the cobalt catalyst and graphene oxide mediator were reused after recovery by centrifugation, while only fresh portions of eosin Y and TEOA were added to restore the reaction system. The observed decrease in activity during repeated catalytic cycles is therefore most likely associated with the gradual photodegradation of eosin Y, a common limitation of dye-sensitized photocatalytic systems. Nevertheless, the cobalt catalyst and graphene oxide mediator retained a large fraction of their activity, indicating good stability of the catalytic components. A key aspect of this study is the evaluation of the photocatalytic system under natural sunlight. Notably, the hydrogen evolution values obtained under natural sunlight are among the highest reported in the literature for comparable systems (Table S1) [20,30,31,32,33,34,35,36,37,38,39], despite the fact that the present photocatalytic system is composed entirely of non-noble elements. These results highlight the strong potential of the Co/GO-based photocatalyst for efficient solar-driven hydrogen production under realistic outdoor conditions. Importantly, analysis combining experimental results with ERA5 meteorological data suggests that high photocatalytic performance is achievable for a substantial portion of the year, with approximately 55% of days reaching at least 80% of the maximum hydrogen production efficiency. This further reinforces the relevance of the investigated system for operation under real outdoor conditions.

In addition to light intensity, factors such as ambient temperature, wind conditions, and transient cloud coverage may contribute to the variability of the measured hydrogen evolution rates. Experiments performed over a wide temperature range revealed that hydrogen evolution remained relatively stable even at temperatures as low as −10 °C. This temperature tolerance is particularly relevant for regions with colder climates and suggest that efficient photocatalytic hydrogen production may be achievable even in northern or temperate regions where ambient temperatures are significantly lower than those typically used in laboratory studies.

These observations indicate that the investigated photocatalytic system maintains high activity despite naturally fluctuating environmental conditions. Such robustness is a key requirement for the practical implementation of solar-driven hydrogen production technologies operating outside controlled laboratory environments.

Overall, the results provide insight into the operational stability and environmental adaptability of dye-sensitized photocatalytic systems under realistic conditions. The ability to achieve efficient hydrogen evolution under moderate sunlight and across a wide temperature range indicates promising prospects for practical implementation in European regions.

## 4. Materials and Methods

### 4.1. Chemicals

Eosin Y (EY), cobalt(II) sulfate heptahydrate (CoSO_4_·7H_2_O), triethanolamine (TEOA) were commercially obtained from Sigma-Aldrich (Merck KGaA, St. Louis, MO, USA) and graphene oxide (GO) was purchased from Layer One. All reagents were used without further purification for the photocatalytic experiments. Solutions and dispersions were prepared using ultrapure water (18 MΩ·cm).

### 4.2. Materials Characterization

Optical absorption properties were assessed by UV–Vis spectroscopy performed on aqueous dispersions of the samples, with spectra collected in the 200–800 nm range using an Agilent Cary 100 spectrophotometer (Agilent Technologies, Santa Clara, CA, USA). Data acquisition and initial processing were performed using Cary WinUV software (v6.6). Chemical functionalities present on the GO surface were identified by Fourier-transform infrared (FT-IR) spectroscopy. FT-IR measurements were carried out using a Bruker IFS 66v/S 161 spectrometer (Bruker Corporation, Billerica, MA, USA) over the wavenumber range of 4000–400 cm^−1^ (sample as tablet with KBr). FT-IR data were processed using Bruker OPUS software (9.0 SP1). The morphological and surface features of GO were examined using atomic force microscopy (AFM). Samples for AFM analysis were prepared by depositing diluted aqueous suspensions onto freshly cleaved mica substrates, followed by drying. AFM images were acquired using an Agilent 5500 atomic force microscope (Agilent Technologies, Santa Clara, CA, USA), and image processing was performed using Gwyddion software (2.70). Vibrational properties of GO were assessed via micro-Raman spectroscopy using an inVia Raman microscope by Renishaw (Wotton-under-Edge, Gloucestershire, UK), equipped with a 50× objective (Leica Microsystems, Wetzlar, Hesse, Germany), operating at a wavelength of λ = 514 nm. Raman spectra were processed using Renishaw Wire software (WiRE 5.5). All experimental data were further processed and analyzed using OriginLab software (2025b(10.25)SR1) (OriginLab Corporation, Northampton, MA, USA).

### 4.3. Photocatalytic Hydrogen Evolution Reaction

The experiments were carried out using an aqueous TEOA solution with a concentration of 0.2 M, which served as a sacrificial electron donor. EY was dissolved in the TEOA solution, yielding a final photosensitizer concentration of 0.5 mM. A separate aqueous solution of CoSO_4_·7H_2_O was prepared at a concentration of 6 mM. In addition, a graphene oxide dispersion was prepared by dispersing graphene paste in water at a concentration of 0.4 mg mL^−1^, followed by sonication for 1 h to ensure efficient exfoliation of the GO flakes.

The individual components were combined in a volumetric ratio of 28:1:1 for the eosin Y, cobalt sulfate, and graphene oxide solutions, respectively, to form the 60 mL reaction mixture. The photocatalytic experiments were carried out in a 100 mL glass bottle equipped with a septum-sealed cap, which served as the photoreactor. Prior to irradiation, the solution was purged with argon to remove dissolved oxygen and continuously stirred throughout the experiment. The photocatalytic reaction was conducted under natural solar irradiation for 1 h, while the light intensity was monitored using a lux meter to record illumination conditions. The overall experimental setup is schematically illustrated in Figure S3. The amount of hydrogen evolved during the reaction was quantified using a gas chromatograph (Agilent 7890B GC Agilent Technologies, Santa Clara, CA, USA) equipped with a thermal conductivity detector (TCD).

In addition to solar-driven experiments, control measurements were performed under three artificial light sources. Photocatalytic experiments under artificial light were carried out using three irradiation sources: a Thorlabs OSL2 high-intensity fiber optic illuminator (Thorlabs Inc., Newton, NJ, USA) equipped with a 150 W halogen lamp, a Thorlabs M505L4 LED source (Thorlabs Inc., Newton, NJ, USA), and a Newport Oriel 6255 ozone-free xenon lamp (150 W) equipped with a cut-off filter blocking wavelengths below 400 nm (Newport Corporation, Irvine, CA, USA). In all cases, the light source was positioned 25 cm from the photoreactor. The irradiance of each source was determined as the incident irradiance at the reactor position using an optical power meter, with the measurements performed under the same source-to-reactor distance and irradiation geometry as those used in the photocatalytic experiments. The measured incident irradiance values were 75 W m^−2^ for the halogen lamp, 150 W m^−2^ for the LED source, and 450 W m^−2^ for the xenon lamp. The reaction mixture was maintained at 25 °C under vigorous stirring. The recyclability of the catalyst material was evaluated by performing three consecutive catalytic runs. Fresh EY/TEOA aqueous solution was replaced after each cycle, followed by flushing the catalytic system with argon and measuring the produced hydrogen at time intervals.

## Data Availability

The original contribution data presented in this research are included in this article: further inquiries can be directed to the corresponding authors.

## Supplementary Materials

The following supporting information can be downloaded at: https://www.mdpi.com/article/10.3390/ijms27093822/s1.
