# Supplementary material for: Photocatalytic Hydrogen Production Driven by Solar Energy: Performance Under Central European Climatic Conditions
Source: Int J Mol Sci. 2026 Apr 25;27(9):3822. doi: 10.3390/ijms27093822 (PMC13163696; doi:10.3390/ijms27093822)
Supplement: Supplementary file 1 [file ijms-27-03822-s001.zip › ijms-4255656-supplementary.pdf]

Supporting Information

Photocatalytic Hydrogen Production Driven by Solar Energy: Performance under Central European Climatic Conditions

Wiktoria Kluba [1], Karol Hauza [1,2], and Anna Lewandowska-Andraloc[1*]

[1] Faculty of Chemistry, Adam Mickiewicz University, Uniwersytetu Poznanskiego 8, 61-614 Poznan, Poland

[2] Center for Advanced Technologies, Adam Mickiewicz University, Uniwersytetu Poznanskiego 10, 61-614 Poznan, Poland

*Correspondence: alewand@amu.edu.pl

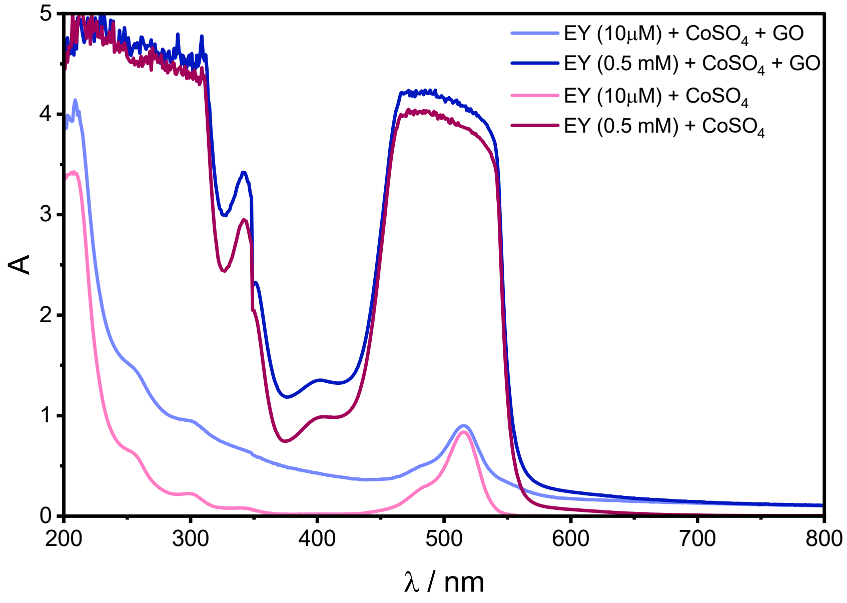

Figure S1. Absorption spectra of the complete reaction mixtures (EY–$CoSO_4$–TEOA) in the presence and absence of GO. Conditions: [EY] = 10 μM / 0.5 mM, [Co^{2+}] = 0.7 mg, [TEOA] = 0.2 M, [GO] = 0.8 mg.

UV–Vis spectra were recorded for eosin Y at two different concentrations: 10 μM and 0.5 mM. The lower concentration corresponds to the conditions used for the individual component spectra, allowing evaluation of the cumulative absorbance and potential shielding effects introduced by GO. At the higher concentration, corresponding to the actual experimental conditions, the absorbance exceeds the measurable range of the spectrophotometer (A > 3) indicating very strong light absorption. Furthermore, absorbance values above 1 are observed below approximately 554 nm, covering the majority of the UV–Vis spectrum. These results clearly indicate that eosin Y is the dominant component responsible for light absorption in the studied system.

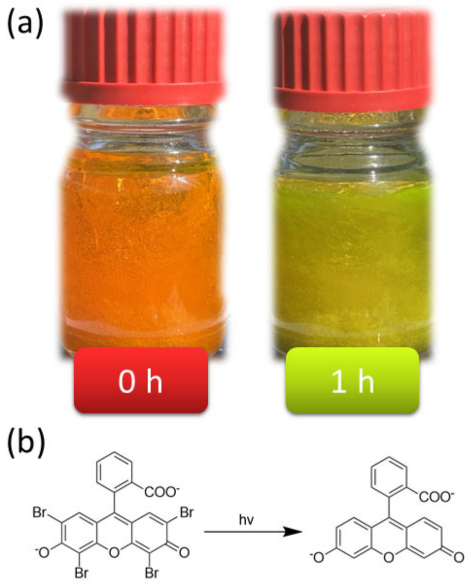

Figure S2. (a) Photographs illustrating the color change of the reaction mixture before and after irradiation. (b) Schematic illustration of the proposed reaction mechanism.

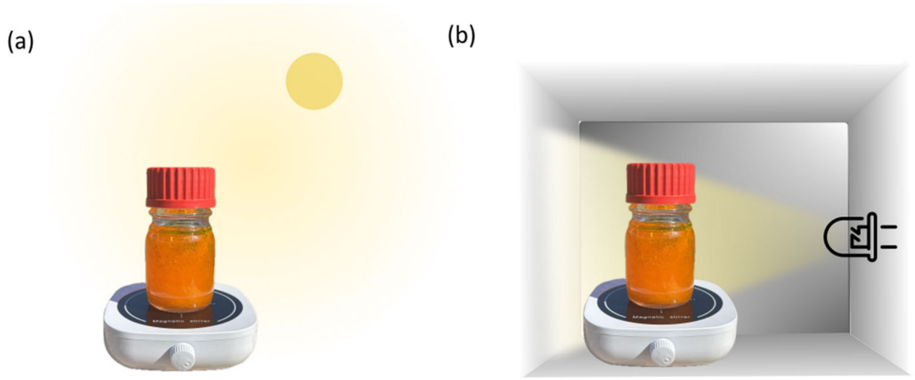

Figure S3. Schematic illustration of the experimental setups used for photocatalytic measurements: (a) outdoor setup under natural solar irradiation, performed using a portable magnetic stirrer powered by a power bank, with the reaction vessel placed at a fixed location and continuously stirred; (b) indoor setup with an artificial light source, where the reaction vessel was placed inside a reflective chamber, and the light source was positioned at a distance of 25 cm from the bottle at the height corresponding to the middle of the solution.

Table 1. Summary of selected literature reports on photocatalytic hydrogen production systems, including EY-based and other representative systems, provided for comparison with the performance of the present system.

Catalyst	Conditions	Hydrogen generation rate	Reference
Graphene QDs/TiO_2	Natural solar light	29.55 mmol g^{-1} h^{-1}	[1]
CdS QDs/thiolated graphene	300 W Xe lamp (> 420)	29.65 mmol g^{-1} h^{-1}	[2]
EY-NiSx/Graphene	300 W Xe lamp (≥ 420 nm)	12.38 mmol g^{-1} h^{-1}	[3]
EY-MoS_2	300 W Xe lamp (≥ 420 nm)	35 mmol g^{-1} h^{-1}	[4]
g-C3N4-TiO_2/rGO	250 W Xe lamp	23.1 mmol g^{-1} h^{-1}	[5]
1T MoS2	100 W halogen lamp	26 mmol g^{-1} h^{-1}	[6]
EY/$Ti_3C_2T_x$/Co^{2+}	λ = 505nm	40.1 mmol g^{-1} h^{-1}	[7]
TiO_2/ Ti_3C_2/carbon nitride nanosheet	λ > 400 nm	15.29 mmol h^{-1} g^{-1}	[8]
$Ti_3C_2T_x$/O-doped g–C_3N_4	λ = 405 nm	25.1 mmol h^{-1} g^{-1}	[9]
NP@COF	simulated sunlight	527.2 ~ 3180.7 mmol h^{-1} g^{-1}	[10]
Ni_2P/CdS NRs	λ = 450 nm	1200 mmol h^{-1} g^{-1}	[11]

References

[1] A. Raghavan, S. Sarkar, L. R. Nagappagari, S. Bojja, S. Muthukondavenkatakrishnan, and S. Ghosh, "Decoration of Graphene Quantum Dots on TiO2 Nanostructures: Photosensitizer and Cocatalyst Role for Enhanced Hydrogen Generation," *Ind. Eng. Chem. Res.*, vol. 59, no. 29, pp. 13060–13068, Jul. 2020, doi: 10.1021/acs.iecr.0c01663.

[2] Z. Fang *et al.*, "Immobilizing CdS quantum dots and dendritic Pt nanocrystals on thiolated graphene nanosheets toward highly efficient photocatalytic H2 evolution," *Nanoscale*, vol. 5, no. 20, pp. 9830–9838, Sep. 2013, doi: 10.1039/c3nr03043a.

[3] C. Kong, S. Min, and G. Lu, "Dye-Sensitized NiSx Catalyst Decorated on Graphene for Highly Efficient Reduction of Water to Hydrogen under Visible

Light Irradiation," *ACS Catal.*, vol. 4, no. 8, pp. 2763–2769, Aug. 2014, doi: 10.1021/cs5006844.

[4] M. Yin, C. Wu, F. Jia, L. Wang, P. Zheng, and Y. Fan, "Efficient photocatalytic hydrogen production over eosin Y-sensitized MoS2," *RSC Adv.*, vol. 6, no. 79, pp. 75618–75625, Aug. 2016, doi: 10.1039/c6ra14710k.

[5] H. Y. Hafeez *et al.*, "Construction of ternary hybrid layered reduced graphene oxide supported g-C3N4-TiO2 nanocomposite and its photocatalytic hydrogen production activity," *Int. J. Hydrogen Energy*, vol. 43, no. 8, pp. 3892–3904, Feb. 2018, doi: 10.1016/j.ijhydene.2017.09.048.

[6] U. Maitra, U. Gupta, M. De, R. Datta, A. Govindaraj, and C. N. R. Rao, "Highly effective visible-light-induced H2 generation by single-layer 1T-MoS2 and a nanocomposite of few-layer 2H-MoS 2 with heavily nitrogenated graphene," *Angew. Chem. Int. Ed.*, vol. 52, no. 49, pp. 13057–13061, Dec. 2013, doi: 10.1002/anie.201306918.

[7] M. Smirnova, B. Scheibe, R. Ramírez-Grau, H. García, and A. Lewandowska-Andralojc, "Synergistic effects of MXene support and cobalt salts in dye-sensitized photocatalytic hydrogen generation," *Int. J. Hydrogen Energy*, vol. 88, pp. 1098–1107, Oct. 2024, doi: 10.1016/j.ijhydene.2024.09.264.

[8] H. Zeng *et al.*, "Interfacial Engineering of TiO2/Ti3C2 MXene/Carbon Nitride Hybrids Boosting Charge Transfer for Efficient Photocatalytic Hydrogen Evolution," *Adv. Energy Mater.*, vol. 12, no. 1, p. 2102765, Jan. 2022, doi: 10.1002/aenm.202102765.

[9] P. Lin, J. Shen, X. Yu, Q. Liu, D. Li, and H. Tang, "Construction of Ti3C2 MXene/O-doped g-C3N4 2D-2D Schottky-junction for enhanced photocatalytic hydrogen evolution," *Ceram. Int.*, vol. 45, no. 18, pp. 24656–24663, Dec. 2019, doi: 10.1016/j.ceramint.2019.08.203.

[10] W. Si *et al.*, "Solar-driven fast photocatalytic hydrogen evolution using size-minimized organic heterojunctions," *Nature Communications 2025 17:1*, vol. 17, no. 1, pp. 1052-, Dec. 2025, doi: 10.1038/s41467-025-67811-4.

[11] Z. Sun, H. Zheng, J. Li, and P. Du, "Extraordinarily efficient photocatalytic hydrogen evolution in water using semiconductor nanorods integrated with crystalline Ni2P cocatalysts," *Energy Environ. Sci.*, vol. 8, no. 9, pp. 2668–2676, Aug. 2015, doi: 10.1039/c5ee01310k.
